# Nano-mechanical properties of Fe-Mn-Al-C lightweight steels

**DOI:** 10.1038/s41598-018-27345-w

**Published:** 2018-06-13

**Authors:** Alireza Rahnama, Hiren Kotadia, Samuel Clark, Vit Janik, Seetharaman Sridhar

**Affiliations:** 1 AI Manufacturing Solutions, 1 Sandover House, 124 Spa Road, London, SE16 3FD United Kingdom; 2 Advanced Steel Research Centre, WMG, University of Warwick, Coventry, CV4 7AL United Kingdom; 3 School of Materials, University of Manchester, Manchester, M13 9PL United Kingdom; 4 Institute for Future Transport and Cities, Coventry University, Coventry, CV1 5FB United Kingdom; 5 George S. Ansell Department of Metallurgical and Materials Engineering, Colorado School of Mines, Golden CO, 80401 USA

## Abstract

High Al Low-density steels could have a transformative effect on the light-weighting of steel structures for transportation. They can achieve the desired properties with the minimum amount of Ni, and thus are of great interest from an economic perspective. In this study, the mechanical properties of two duplex low-density steels, Fe-15Mn-10Al-0.8C-5Ni and Fe-15Mn-10Al-0.8 C (wt.%) were investigated through nano-indentation and simulation through utilization of *ab-initio* formalisms in Density Functional Theory (DFT) in order to establish the hardness resulting from two critical structural features (*κ*-carbides and B2 intermetallic) as a function of annealing temperature (500–1050 °C) and the addition of Ni. In the Ni-free sample, the calculated elastic properties of *κ*-carbides were compared with those of the B2 intermetallic Fe3Al−L1_2_ and the role of Mn in the *κ* structure and its elastic properties were studied. The Ni-containing samples were found to have a higher hardness due to the B2 phase composition being NiAl rather than FeAl, with Ni-Al bonds reported to be stronger than the Fe-Al bonds. In both samples, at temperatures of 900 °C and above, the ferrite phase contained nano-sized discs of B2 phase, wherein the Ni-containing samples exhibited higher hardness, attributed again to the stronger Ni-Al bonds in the B2 phase. At 700 °C and below, the nano-sized B2 discs were replaced by micrometre sized needles of *κ* in the Ni-free sample resulting in a lowering of the hardness. In the Ni-containing sample, the entire α phase was replaced by B2 stringers, which had a lower hardness than the Ni-Al nano-discs due to a lower Ni content in B2 stringer bands formed at 700 °C and below. In addition, the hardness of needle-like *κ*-carbides formed in α phase was found to be a function of Mn content. Although it was impossible to measure the hardness of cuboid *κ* particles in γ phase because of their nano-size, the hardness value of composite phases, e.g. γ + *κ* was measured and reported. All the hardness values were compared and rationalized by bonding energy between different atoms.

## Introduction

In order to reduce energy consumption and greenhouse gas emissions in the next generation automobiles, there is an increasing demand for the development of advanced high-strength steels with high strength to weight ratio^1,2^. With excellent mechanical properties^3–8^, Fe-Mn-Al-C low-density steels are promising candidates for such applications. These steels were originally designed to replace the stainless steels because of their good oxidation and corrosion resistance but recently they have regained interest because of the significant reduction in mass density due to alloying with Al, and are therefore considered as promising candidate materials for an automotive body in white^3,5^.

Their success depends on the design of the alloy composition and processing route to enhance the precipitation of various ordered nano-sized particles. Depending on the Al content, various types of ordered structures can form in the microstructure of low-density steels, i.e. B2, L1_2_, ${\rm{L}}{1^{\prime} }_{2}$, etc. B2 has a bcc structure where the Fe (Ni) atoms are positioned at the corners of the unit cell and the Al atom occupies the central position. ${\rm{L}}{1^{\prime} }_{2}$ is a perovskite structure with the Fe_3_AlC formula that is commonly denoted as a *κ*-carbide phase. The addition of Mn into this carbide structure leads to the formation of Fe_2_MnAlC, FeMn_2_AlC and Mn_3_AlC phases. These phases have a structure similar to the ordered fcc phase, Fe_3_Al-L1_2_, wherein the Fe atoms are located at the centre of each face and the Al atoms at the corners of the unit cell. The additional carbon atom to this structure results in ${\rm{L}}{1^{\prime} }_{2}$ where carbon occupies the central octahedral interstitial position formed by the six Fe atoms as its nearest neighbours. These precipitates are formed by the ordering of alloying elements such as C, Mn, and Al^9–11^.

The morphology of the ordered precipitates were engineered in order to improve the properties of low-density steels^12,13^, and various experimental studies show that the overall properties of the bulk material strongly depends on the size and morphology of ordered phases^14^. The characteristics of ordered phases are determined by their degree of coherency with the matrix within which they form^9^ and the strength of these ordered phases. Consequently, many investigations show that the stiffness of the bulk material depends on the composition of the ordered phases, for example, an increase in the Mn content of the *κ*-phase can significantly increase the strength of the bulk material^15^. This effect was attributed to the substitution of weaker Fe-C bonds with stronger Mn-C bonds. Thus, while engineering the structure of these steels, it is of importance to determine the properties of individual ordered phases and their contribution to the bulk mechanical properties of the low-density steels.

**Figure 1:**
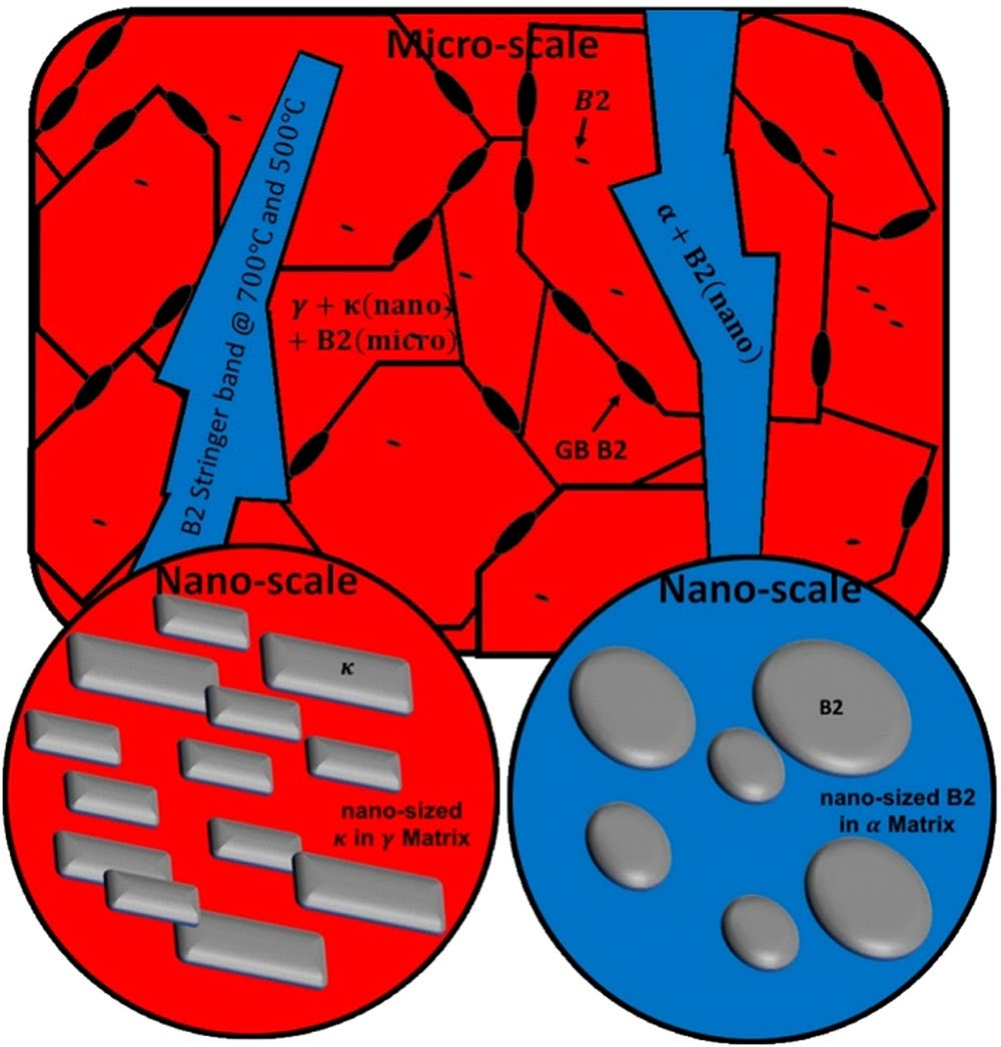
The schematic representation of ordered precipitates observed in the microstructures of steels under study.

**Figure 2:**
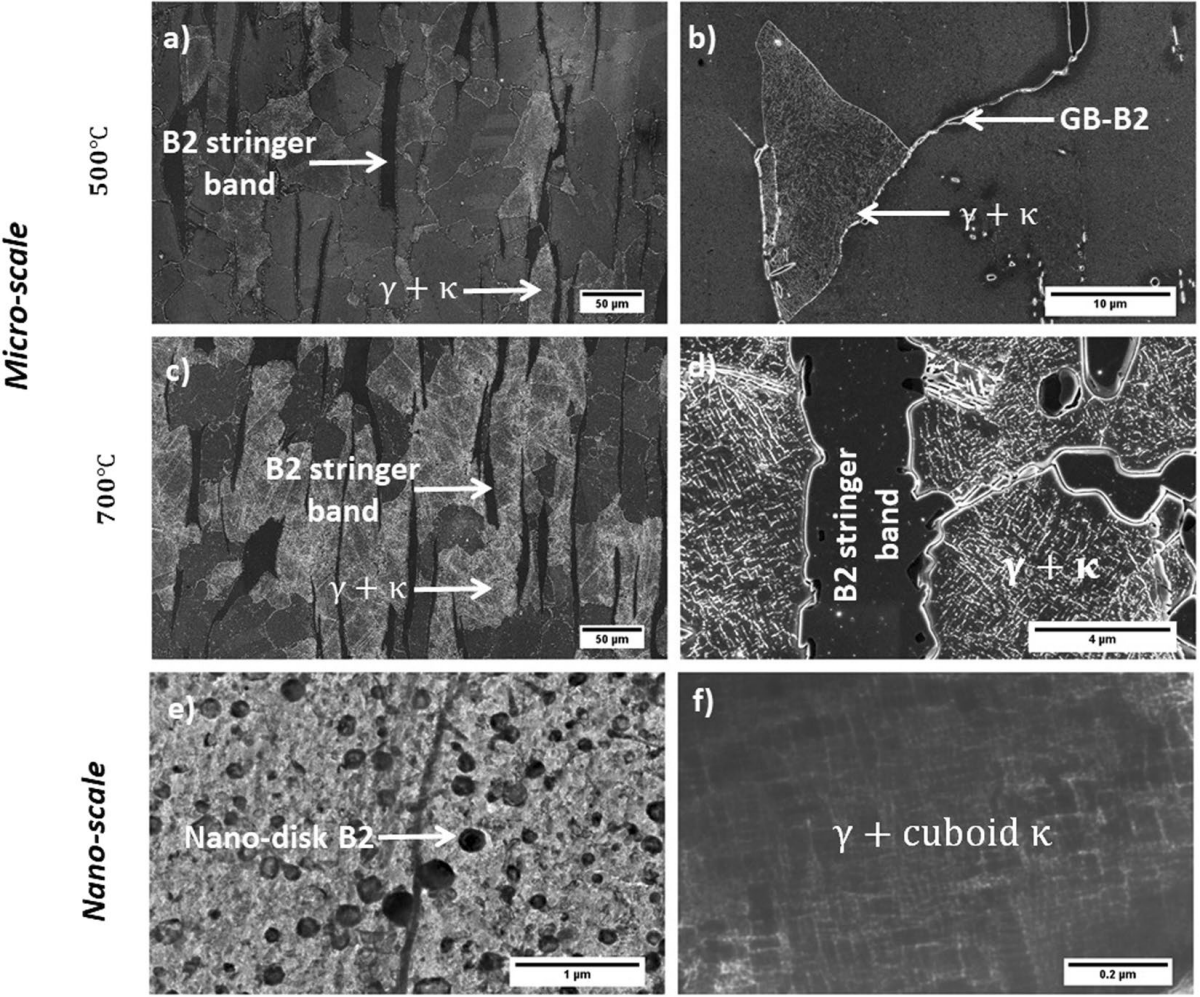
Microstructural variation of low-density steels with annealing temperature. (**a**) SEM micrograph showing the formation of nano-sized ***κ***-carbidess in the interior of ***γ*** grains, and the formation of B2 stringer bands (initially α phase). (**b**) The formation of coarse grain boundary B2 particles (GB-B2). (**c**) and (**d**) Similar microstructure to the one formed during annealing at 500 °C was observed after isothermal holding at 700 °C. (**e**) TEM-bright field image of a disk-like B2 particle in the α matrix after annealing at higher temperatures (e.g. 900 °C and 1050 °C). (**f**) TEM-bright field image of cuboid κ particles in the ***γ*** matrix. All micrographs show the microstructure of Ni-containing sample. The Ni-free followed the similar trend except that at lower annealing temperatures (e.g. 500 °C and 700 °C) where α + needle-like κ-carbides were observed instead of B2 stringer bands.

Our previous study^11^ mapped out the phases present in two duplex low-density steels, one with 5 wt.% Ni and the other being Ni-free, in the temperature range of 500 to 1050 °C. For both the grades at temperatures from 900 to 1050 °C^9–11^ B2 formed as micron-sized particles and grain boundary precipitates in the austenite phase and as nano-sized discs in the ferrite (see Fig. 1 (blue circle) and Fig. 2b and e). The discs were of Ni-Al type in the Ni-containing samples but of Fe-Al type in Ni-free samples. At temperatures from 500 to 700 °C, *κ*-carbides formed nano-sized blocks in the austenite phase in both Ni-containing and Ni-free samples and as micron-sized needles within the ferrite only in Ni-free sample (Fig. 1 (red circle) and Fig. 2b and f). At low annealing temperature between 500 and 700 °C, the ferrite phase in the 5% Ni sample was found to be replaced by B2 stringer bands. All ordered structures observed in the microstructures of the steels are summarized in Table 1.

**Table 1:** Phases present in the microstructure of 5 wt.% Ni and Ni-free samples as a function of annealing temperature.

Alloy		500 °C		700 °C		900 °C		1050 °C	
Fe-15Mn-10Al-0.5C-5Ni	Matrix Phase	*γ*		*γ*		*γ*	*α*	*γ*	*α*
	Precipitates	Cuboidal *κ* ( < 100 nm) (•)		Cuboidal *κ* ( < 100 nm) (•)		Coarse B2 Particles (2–7 μm) Matrix: (♦)	Disc-like B2 (≈200 nm) (♦)	Coarse B2 Particles (2–4 μm) (♦)	Disc-like B2 (≈200 μm) (♦)
		Coarse B2 Particles (2–7 μm) (♦)		Coarse B2 Particles (2–7 μm) (♦)					
		B2 Stringer Bands (♣)		B2 Stringer Bands (♣)					
Fe-15Mn-10Al-0.5 C	Matrix Phase	*γ*	*α*	*γ*	*α*	*γ*	*α*	*γ*	*α*
	Precipitates	Coarse B2 Particles (2–4 μm) (♦)	Needle-like *κ* (width 200 ± 33 nm) (♠)	Coarse B2 Particles (2–4 μm) (♦)	Needle-like *κ* (width 500 ± 33 nm) (♠)	Coarse B2 Particles (2–4 μm) (♦)	Disc-like B2 (≈200 nm) (♦)	Coarse B2 Particles (2–4 μm) Matrix: (♦)	Disc-like B2 (≈200 nm) (♦)
		Needle-like *κ* (width 200 ± 33 nm) (♠)		Needle-like *κ* (width 500 ± 33 nm) (♠)					

Our previous study showed, moreover, that at lower annealing temperatures, the materials had low ductility (elongation ≈10%). Increasing the annealing temperature led to significant improvement of the strength-ductility balance in both the grades due to the removal of *κ*-carbides and the formation of nano-sized B2 disks in the ferrite phase^11^. This study also demonstrated that the room temperature mechanical properties of the samples remarkably varied with any change in the composition of precipitates. These findings clearly showed that the determination of the properties of nano and micron-sized precipitates is of scientific and technological importance and can be utilized for the future developments of low-density steels. This scientific knowledge and experimental trends were used as the basis for further computational modelling efforts to explain the differences in properties based on precipitation composition and type.

The aim of the present work is to evaluate the nanomechanical properties of each individual ordered phase and of the matrix as a function of annealing temperature and the precipitate composition. Also, the nano-mechanical composite properties of the combined ordered precipitates and the matrix were determined. Because it is technically not possible to determine the properties of individual nano-sized blocks of *κ*-carbides through nano-indentation, we carried out a series of first principle calculations to determine the elastic constants and thereby the Young’s modulus of nano-sized cuboid *κ*-phase formed in austenite as a function of the Mn content.

## Materials and Methods

### Materials

Two duplex lightweight steels Fe-15Mn-10Al-0.8C-5Ni and Fe-15Mn-10Al-0.8 C (all in wt.%) were investigated in the presented work. These specific grades of steel were selected to determine and compare the nanomechanical properties of the ordered NiAl and FeAl-type B2 compounds and the *κ*-phase (${\rm{L}}{1^{\prime} }_{2}$), which formed in both grades. The experimental alloys were melted in an induction furnace, solution heat-treated for 35 *min* at 1250 °C in a protective argon atmosphere and then water quenched. The materials were then cut into 3-*mm* (length), 3-*mm* (width) and 1*-mm* (thickness) samples. The annealing treatments were conducted at 500 °C, 700 °C, 900 °C, and 1050 °C. The highest annealing temperature of 1050 °C was selected to study the change in the nanomechanical properties arising from an increase in the Ni and Al content of the nanosized disk-like B2 particles.

### Characterization

The ordered phases were characterized by examination of selected-area diffraction patterns (SADPs) and bright-field (BF) images in transmission electron microscopy (TEM) JEOL 2000FX. Thin foils for TEM observation were prepared by twin-jet electro-polishing in a mixture of 10% perchloric acid and 90% ethanol with an applied potential of 25 *V*. The steel specimens were, also, polished and etched in a 5% nital solution and the microstructures were observed using a Carl Zeiss Sigma Field Emission Scanning Electron Microscope (FE-SEM) operated at 20 kV. The equipment was fitted with high-speed X-ray energy dispersive spectroscopy (EDS).

### Nano-indentation

**Figure 3:**
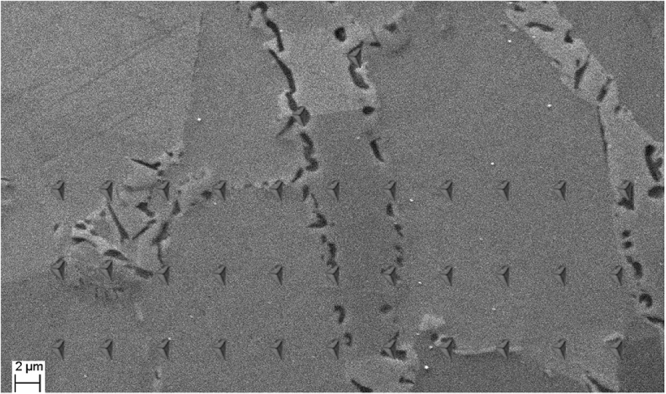
Rows of nano-indentations carried out to determine the nano-hardness of different phases present in the microstructure.

Nano-indentation tests were carried out at a constant loading/unloading rate of 500 *μNs*^−1^ up to a maximum load of 10000 *μN* with a hold time of 20*s*. Each measurement in a particular phase was repeated at least 25 times and in arrays of 50−70 indentations applied across the region of interest. The measurements were performed for the following individual phases: solution-treated *γ* and *α* phases, B2 stringer bands, and both micro-meter sized and nano-disk B2. The hardness of the following combinations of phases was also determined because either it was impossible to measure the value of each phase individually or the combined hardness values were believed to be of interest for the future studies: *γ* + cuboid *κ*, *γ* + cuboid *κ* + microsized B2, *γ* + microsized B2, *α* + needle-shape *κ*, and *α* + nano-disk B2. An example of nano-indentation indents as recorded by SEM is shown in Fig. 3.

### *ab-initio* calculations

DFT calculations were carried out on different ordered phases using the method proposed by Hohenberg and Kohn^16,17^. The generalized gradient approximation (GGA) of Perdew-Burke-Ernzerhof (PBE)^18^ in its spin-polarized manner was employed for the exchange and correlation functional. Calculations were performed using the Quantum-*ESPRESSO* package^19,20^. Ultrasoft pseudo-potentials^21^ were created based on a modified Rappe-Rabe-Kaxiras-Joannopoulos (RRKJ) scheme according to the method of Rappe *et al*.^22^. For structure optimization, the Brillouin zone (BZ) integrations were done by an 18 × 18 × 18 Monkhorst and Pack grid^23^. A real-space grid with a 50 *Ryd* energy cut-off was used for the calculations of the charge density. The augmentation charges were expanded up to 600 *Ryd*. A 30 × 30 × 30 grid with a 60 *Ryd* energy cut-off was used for the electronic density of states and the formation energy calculations. To calculate the bulk modulus, various unit cell volumes were simulated, and the values were fitted using a Murnaghan equation of state^24^. Dynamical matrices were deduced by the linear response of the electronic subsystem in the harmonic approximation. The density of states was calculated by the tetrahedron method with a 30 × 30 × 30 mesh.

## Results and Discussion

### Experimental results

**Figure 4:**
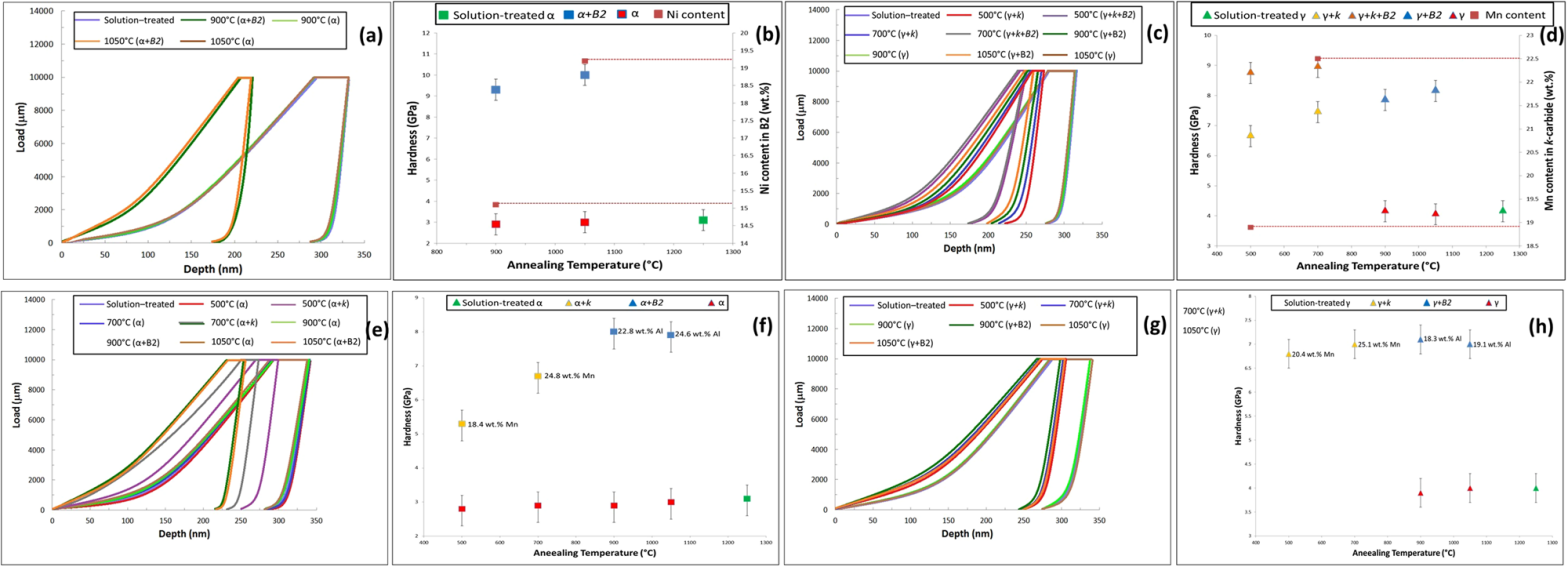
(**a**) The load-depth curves of ***α*** phase and its composites in Ni-containing sample and (**b**) the corresponding hardness values. (**c**) The load-depth curves of austenite and its composites in Ni-containing sample and (**d**) their corresponding hardness values. (**e**) The load-depth curves of ***α*** and its composites in Ni-free sample and (**f**) the corresponding hardness values. (**g**) The load-depth curves for ***γ*** phase and its composites in Ni-free sample and (**h)** their corresponding hardness values.

Nano-indentation test results have been observed as function of four annealing temperatures (500 °C, 700 °C, 900 °C and 1050 °C) and two alloys compositions (Fe-15Mn-10Al-0.8C-5Ni and Fe-15Mn-10Al-0.8 C (all in wt.%)). Figure 4 shows the load-depth curves along with corresponding hardness values for the bulk *α* and *γ* phases (without precipitates) as well as the composite bulk phases containing differently ordered precipitates. It is important to note that the nanoindentation technique as performed in this study is unable to measure the hardness of isolated nano-sized precipitates, but measures instead the hardness of the nano-sized features within a surrounding matrix, and therefore yield a compound hardness value of the matrix with particles. In the present study, we observed two B2 phase with distinct morphology: B2 with flat interfaces and sharp edges with an average size of ≈1.4 μm formed in the γ-phase and disk-like precipitates with a size of a few hundred nanometres formed in α-phase. Additionally, κ -carbide formed in the γ phase had a cuboid shape with sizes of less than 100 nm and those formed in α-phase had a needle-like shape with an average size of ≈ 1.5 μm.

The measured hardness values of various phases are all summarized in Table 2. The basic trends from Fig. 4 and Table 2 are listed below for the *α* and *γ* phases. The results for the hardness of the *α* phase and the composites formed with the *α* phase were found to be as follows:

1. The hardness value of *α* was approximately 3 GPa in the solution-treated materials (Ni and Ni-free).
2. The hardness values for the Ni-free sample were measured to be 5.3 *GPa* for *α* + needle-shape *κ* in the sample annealed at 500 °C and 6.7 *GPa* for *α*+ needle-shape *κ* in the sample annealed at 700 °C. *κ*-particles precipitated at 700 °C contained higher Mn (≈24.8 *wt*.%) compared to those formed at 500 °C(≈18.4 *wt*.%) meaning that a higher annealing temperature affects the formation energy, lattice parameter and magnetization of *κ*-phase resulting in the formation of harder *κ* particles. This was confirmed by our *ab-initio* calculations which are discussed later in this paper. At higher temperatures, when the precipitates changed to FeAl-type B2, the hardness increased to 8.9 *GPa* for *α* + B2 and 8.3 *GPa* in the samples annealed at 900 °C and 1050 °C Al content of the particles ranged from 22.8 to 24.6 *wt*.%.
3. For the Ni-containing samples, the precipitates changed for all annealing temperatures to NiAl-type B2 and the measured hardness values were for the composite structures: 11.7 *GPa* for *α* + B2 for samples annealed at 900 °C, and13.5 *GPa* for *α* + B2 for samples annealed at 1050 °C. At lower temperatures, the B2 precipitates changed to stringer bands and resulted in hardness values of 10.1 *GPa* for samples annealed at 500 °C and 10.6 *GPa* for stringer bands annealed at 700 °C. It was found that the variation of hardness values of B2 in Ni-containing sample is a function of Ni content. Increasing annealing temperature resulted in an increase in the Ni content of B2 particles which ultimately led to an increment in the hardness values.
  To summarize the results for the hardness of the γ phase and the composites formed with the γ phase:
4. The hardness was found to be approximately 4.2 *GPa* and 4.0 *GPa* for the solution-treated single *γ*-phase in both Ni and Ni-free samples respectively.
5. For the Ni-containing samples the hardness was measured to be 6.7–7.5 *GPa* for *γ* + cuboid *κ* and 8.8 −9*GPa* for the *γ* + cuboid *κ* + B2 composite phase for samples annealed at 500–700 °C respecively. At higher annealing temperatures 900–1050 °C only B2 precipitated and the measured hardness values were 7.9−8.2 *GPa γ* + B2 for the sample annealed at 900 and 1050 °C respecitively.
6. For the Ni-free sample, the hardness values were, 6.8–7 *GPa* for *γ* + cuboid *κ* annealed at 500 °C and 700 °C. At higher annealing temperatures B2 was the only phase to precipitate but the hardness did not change significantly (7.2 GPa for *γ* + B2 in the sample annealed at 900 °C and 7 GPa for the *γ* + B2 in the same sample at 1050 °C.

**Table 2:** Summary of the hardness values for various individual phases and that of the combinations of phases.

Alloy	Temperature °C	Hardness (GPa)							
Fe-15Mn-10Al-0.5C-5Ni	Phases	*γ*	*α*	*γ* + *κ*	*α* + *κ*	*γ* + B2	*α* + B2	B2 Stringer	*γ* + *κ* + B2
	Solution Treated	4.2	3.1	—	—	—	—	—	—
	500	—	—	6.7 (•)	—	—	—	10.1 (♣)	8.8 (•♦)
	700	—	—	7.5 (•)	—	—	—	10.6 (♣)	9 (•♦)
	900	4.1	2.9	—	—	7.9 (♦)	11.7(♦)	—	—
	1050	4	3	—	—	8.2 (♦)	13.5 (♦)	—	—
Fe-15Mn-10Al-0.5 C	Phases	*γ*	*α*	*γ* + *κ*	*α* + *κ*	*γ* + B2	*α* + B2	B2 Stringer	*γ* + *κ* + B2
	Solution Treated	4	3	—	—	—	—	—	—
	500	—	—	6.8 (♠)	5.3 (♠)	—	—	—	—
	700	—	—	7 (♠)	6.7 (♠)	—	—	—	—
	900	3.8	2.9	—	—	7.2 (♦)	8.9 (♦)	—	—
	1050	4	3	—	—	7 (♦)	8.3 (♦)	—	—

The trends listed in 1–6 suggests that the hardness increases in the composite structures resulting from higher temperature annealing, when precipitates are predominantly B2 and the highest hardness is found in the structure disk-like B2 formed in the ferrite (hardness value = 10 *GPa*). According to Orowan mechanism, the increase in shear stress (Δτ) is proportional to the inverse of the precipitates’ diameter: Δτ ∝ R^−1 ^^25^. In other words, as the size of precipitates decreases the resistance to shear increases. Thus, the higher hardness values obtained at higher annealing temperatures can be also attributed to the formation of uniformly distributed nano-sized disk-like B2 particles which increase the shear resistance of the α-matrix^10^. It can be seen that the hardness of γ + (nanosized-κ-carbide) + (microsized-B2) composite reached a higher value than γ + micron sized B2 composite. This is despite the fact that the κ-carbides are known to not be as resistant to shear as B2 intermetallic compounds. Therefore, the addition of brittle nano-sized κ-carbide to γ + micron sized B2 composite increase the hardness further.

**Figure 5:**
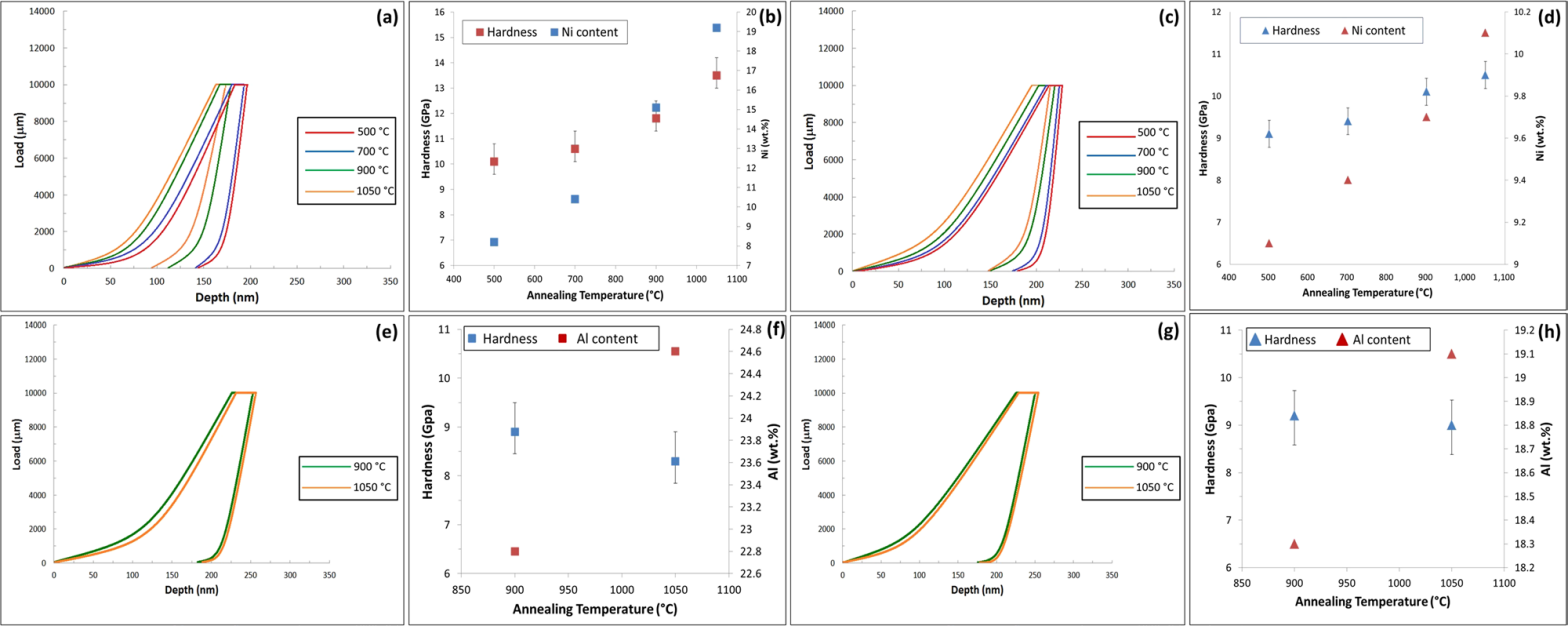
(**a**) The load-depth curves of NiAl-type nanosized disk-like B2 phase formed within an α matrix in the Ni-containing sample for the four annealing treatments and (**b**) the corresponding hardness values. (**c**) The load-depth curves of coarse B2 particles precipitated in the γ matrix in the Ni-containing sample and (d) their corresponding hardness values. (**e**) The load-depth curves of NiAl-type nanosized disk-like B2 phase formed within an α matrix in the Ni-free sample for the four annealing treatments and (**f**) the corresponding hardness values. (**g**) The load-depth curves of coarse B2 particles precipitated in the γ matrix in the Ni-free sample and (**h**) their corresponding hardness values.

The hardness values of the coarse NiAl-type B2 particles precipitated in the *γ* phase of Ni-containing sample was less than those of the corresponding precipitates in the *α* (Fig. 5d). This phenomenon occurs mainly due to the Ni atoms primarily accumulated in the *α* phase to form the B2 stringer bands at the two lower annealing temperatures. At higher annealing temperatures, more Ni atoms were available to form the nanosized-B2 particles in the *α* phase. Therefore, hardness values increase from 9.1 *GPa* (Ni: 8.2 wt.%) at 500 °C to 10.5 *GPa* (Ni: 10.1 *wt*.%) at 1050 °C.

However, for Ni-free sample, the values of coarse FeAl-type B2 particles in the *γ* and nano-sized B2 particles did not differ significantly (Fig. 5h). The values were recorded to be 9.2 *GPa* for annealing at 900 °C (8.9 *GPa* for nanosized B2 in *α* formed during the same treatment) and 9 *GPa* for annealing at 1050 °C (8.3 *GPa* for the same phase in *α* precipitated during the same treatment). The Al content of B2 particles formed in *γ* phase was less than those formed in *α* (Al is an *α* stabiliser) and this was anticipated to lead to an increase in the hardness of the particles. For a detailed discussion on the effects of size and morphology of ordered precipitates on the mechanical properties of low-density steels, the reader is referred to refs^10,11^.

The nanoindentation technique as performed in this study was only able to measure the hardness of the nano-sized features as present within the surrounding matrix, and therefore yields a compound hardness value of the matrix with particles. To evaluate the possible size-effect of the differently sized phases on the hardness of the matrix and particle composite, it is required to perform nanoindentation using different indentors or of different applied loads. Unfortunately, changing the indentation size or volume will further introduce a nano-indentation size-effect as a function of varying size and shape of the plastic zone below the indent^26,27^ which may obscure the real influences of the various precipitates and their sizes on the hardness of the composite microstructure.

### DFT calculations

**Figure 6:**
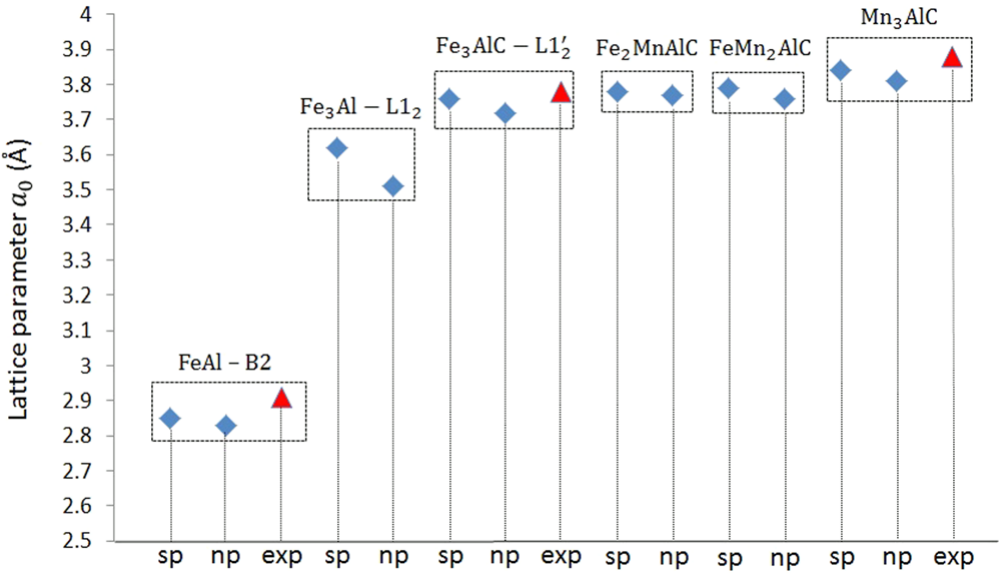
The lattice constants for different ordered compounds: *FeAl-B2*, *Fe*_3_*Al*-*L*1_2_, *Fe*_3_*AlC*-*L*1_2_, *Fe*_2_*MnAlC*, *FeMn*_2_*AlC*, and *Mn*_3_*AlC*. Experimental values are indicated by red triangles.

We performed first-principle calculations to determine the lattice parameter and thus elastic constants of *κ*-carbide. Although, the calculated lattice parameter of the ordered precipitate FeAl-B2 for cases with or without spin effects (spin-polarized (*sp*), without spin effects (*np*), the blue diamond in Fig. 6) was slightly less than the experimental value (red triangle), the values were still in good agreement with the reported experimental ones. The calculations for without spin effects were performed as it was reported in the literature that the perfect FeAl-B2 phase does not exhibit a macroscopic magnetic momentum^28^. Calculations were also carried out for the Fe_3_Al-L1_2_ compound for comparison with the Fe_3_AlC phase. The lattice constant for Fe_3_AlC was calculated to be larger than that for Fe_3_Al-L1_2_ which agrees well with the experimental observations reported in refs^29,30^. The insertion of the C atom in the crystal structure of L1_2_ in the octahedral interstitial site induces an expansion of the lattice parameter. The calculations showed that the substitution of the Fe atoms by the Mn atoms increases the lattice parameters of the structure from 3.78 $\dot{{A}}$ for Fe_2_MnAlC to 3.84 $\dot{{A}}$ for the Mn_3_AlC phase^29,31,32^. This increase in the lattice parameter because of insertion of additional Mn atoms was believed to be due to the larger atomic radii of Mn (*r*_*Mn*_ = 1.61$\dot{{A}}$) compared to that of Fe (*r*_*Fe*_ = 1.56$\dot{{A}}$)^33^.

DFT calculations^34,35^ of the effects of increasing Al on the elastic constants of Fe-Al compounds showed that as the composition changes from FeAl to FeAl_2_ and FeAl_3_ the C_11_ elastic constant decreases from 294 to 223 and 168.5 (all in *GPa*) respectively, while the value of C_44_ reduced from 157 to 109.6 and 76.1 *GPa* when the structure changed from FeAl to FeAl_2_ and FeAl_3_ respectively. On the other hand, for Ni-Al compounds, ab-initio calculations^36^ showed that C_11_ increased from 221.67 to 375.61 *GPa* and C_44_ increased from 116.99 to 133.21 *GPa* by changing the structure from NiAl to Ni_3_Al. From values of elastic constants of the two compounds, Ni-Al compounds have elastic constants with higher values compared to Fe-Al compounds. In addition to that Breuer *et al*.^37^ conducted a series of experiments and measured the enthalpy of formation of B2-Fe_1-x_-Al_x_ and B2-(Fe, Ni)_1-x_-Al_x_ at 1073 K. Their results showed that generally, enthalpy of formation of B2-(Fe, Ni)_1-x_-Al_x_ is of greater magnitude than that of B2-Fe_1-x_-Al_x_. It was also shown that starting with binary B2-Fe_1-x_-Al_x_ and replacing Fe with Ni, B2-(Fe, Ni)_1-x_-Al_x_ while keeping the Al content at a constant value, the enthalpy of formation becomes increasingly more negative^37^. In other words, increasing the Ni content of B2 can result in the formation of a harder B2 phase. This is supported by the experimental results in Table 2 when comparing the composite structures, with Ni vs. without Ni, containing B2 in both austenite and ferrite. Higher annealing temperatures led to an increase in the Ni content of B2 phase. As is obvious from Table 2, the hardness values increased for those B2 particles formed at higher temperatures.

The ferromagnetic and non-magnetic states of ordered Fe_3_AlC were also investigated. The ground state was calculated to be 30*meV*/*at* in favour of the ferromagnetic state with a magnetic moment (1.1*μ*_*B*_/*at*.*Fe*) more than twice smaller than in Fe_3_Al. To study why the magnetic moment decreased compared to the L1_2_ compound, we investigated the role of the lattice parameter on the magnetic moment. A numerical study was performed in which the lattice parameter of Fe_3_AlC was imposed to Fe_3_Al. The results showed that the decrease of the magnetism from L1_2_ to imposed-${\rm{L}}{1}_{2}$ was not due to the increase in the lattice parameter (${\mu }^{L{1}_{2}}({a}_{0}=3.76)=$ $2.39 > {\mu }^{L{1}_{2}}({a}_{0}=3.62)=2.24$). It was therefore assumed that the decrease in the magnetism is due to the interactions between Fe and C atoms. 

**Figure 7:**
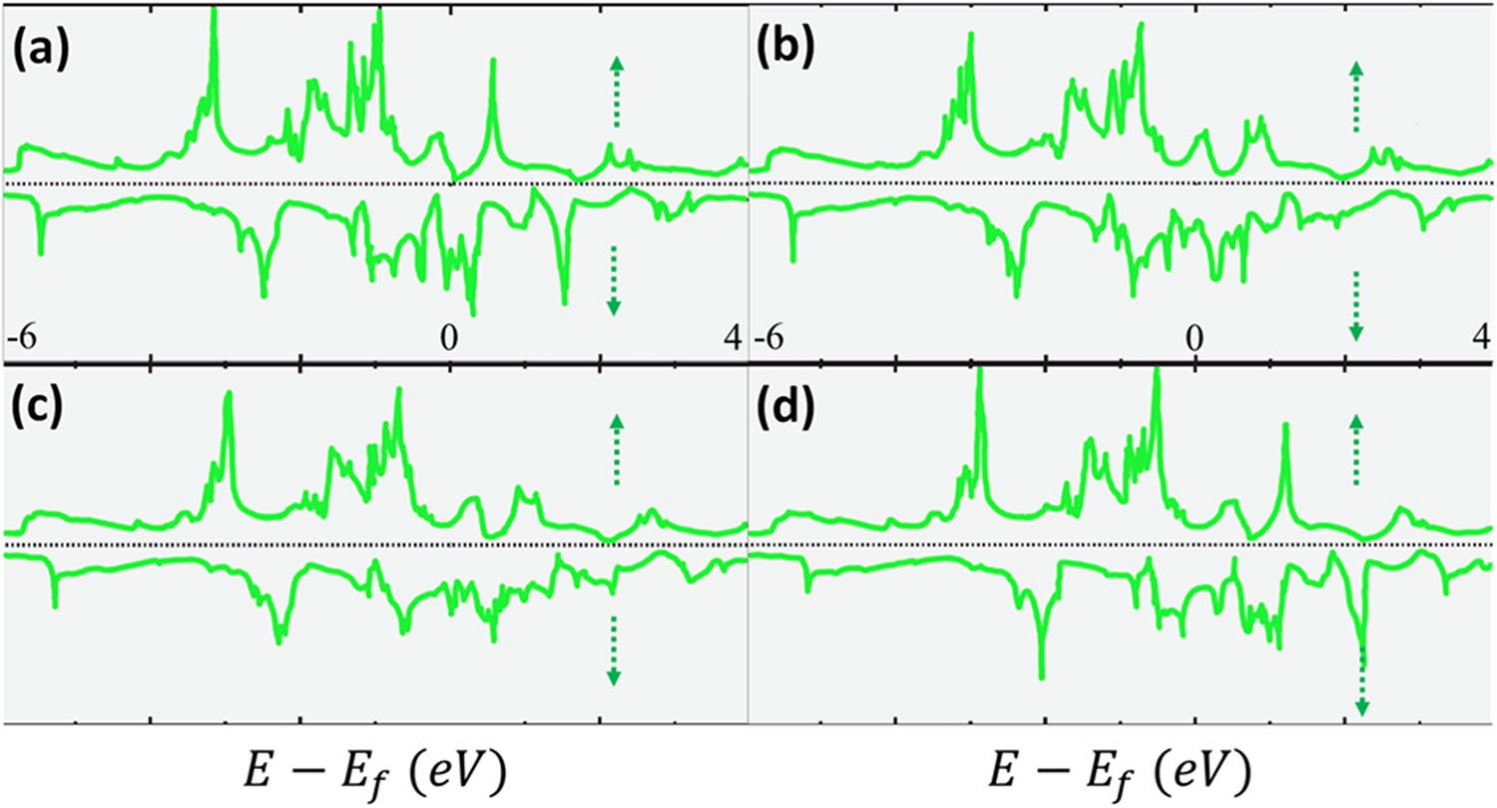
Total density of states of (**a**) Fe_3_AlC, (**b**) Fe_2_MnAlC, (**c**) FeMn_2_AlC, and (**d**) Mn_3_AlC.

The total Density of States (DOS) of the four crystalline  κ-carbides are presented in Fig. 7. The overall shapes of the four structures were very similar. The only difference was the so-called ‘rigid band shift’: the shift of the peaks to higher energy or equivalently the shift of the Fermi level to lower energy. This was believed to result from two effects: first Mn has one less electron than Fe, and second, the replacement of Fe by Mn barely produces a change in the electronic states around the Fermi level.

**Figure 8:**
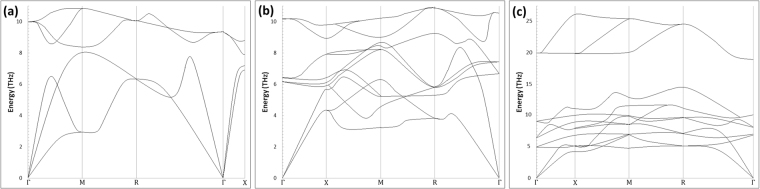
Vibrational band structures for (**a**) *FeAl*, (**b**) ***Fe***_3_***Al***, and (**c**) ***Fe***_3_***AlC*** compound.

The vibrational band structures for FeAl, Fe_3_Al and Fe_3_AlC phases were calculated with the SGGA approximation. In the case of FeAl (Fig. 8a), the high-frequency branches were initiated mainly from the low mass Al atoms whereas the low-frequency bands primarily originated from the high-mass Fe atoms. These two bands were separated by an optical gap. The optical gap (around 0.7 THz) had the same width as that calculated for FeAl-B2 or DO3^38^, while the results on Fe_3_Al-L1_2_ were close to those calculated for Ni_3_Al-L1_2_^39^. As for Fe_3_Al (Fig. 8b), the result showed that the structure is stable, although it was reported in the literature that within the GGA approximation the L1_2_ structure is not mechanically stable^38^. This anomaly between the present calculation and those reported in the literature showed that the properties are very sensitive to the choice of the functional. The vibrational band structure for Fe_3_AlC was shown in Fig. 8c. In contrast to the previous calculation for Fe_3_Al, no significant differences were noted between the present calculations (GGA approach) and those reported in the literature (SGGA approach).

We used two methods to evaluate the elastic constants of different compounds under study:

- The elastic constants *C*_*ij*_ were derived from the vibrational acoustic dispersion curves presented above. For the cubic systems, only three elastic constants, namely *C*_11_, *C*_12_, and *C*_44_, are independent.
- In the second method, a couple of lattice distortions was used to determine the elastic constants^40–42^.

The detailed descriptions of both methods are explained in the methods section.

The calculation results are summarized in Table 3. For Fe_3_Al the elastic constants were similar to other Fe-Al ordered phases. The occupation fo the octahedral interstitial site by C atoms rigidified the Fe_3_AlC in comparison with *Fe*_3_*Al*. This is the direct manifestation of the strong Fe-C bonds in the < 100 directions. The calculations revealed that all elastic constants of Mn_3_AlC are greater than those of Fe_3_AlC confirming the experimental hardness values reported above (the increase in the Mn content of *κ*-carbide resulted in an increase in the hardness value of the phase). This is also a direct consequence of strong Mn-C bonds which are harder than Fe-C bonds. For the tetragonal structures, Fe_2_MnAlC and FeMn_2_AlC, *C*_11_ and *C*_33_ are predominantly greater than the other elastic constants indicating the formation of strong Mn-C bonds in 〈100〉 directions.

**Table 3:** Elastic constants for the compounds under study (in GPa).

Compound	C_11_		C_12_		C_44_		C_13_	C_33_	C_36_
FeAl	273	−293	110	−122	149	−160	—	—	—
Fe_3_Al	181	−184	139	−165	163	−124	—	—	—
Fe_3_AlC(sp)	450	−431	100	−94	73	−68	—	—	—
Fe_2_AlC	—	−427	—	−80	—	−96	−97	−471	−98
FeMnAlC	—	−470	—	−81	—	−100	−143	−451	−101
Mn_3_AlC	—	−448	—	−98	—	−110	—	—	—

The values in parenthesis were calculated using the second method (lattice distortion).

From the calculated set of elastic constants, practical parameters such as bulk modulus *B*_0_, the factor of anisotropy *C*_*a*_, the shear modulus *G*, and the Young’s modulus *E* in two crystallographic directions ([100] and [111]) were calculated (see methods) and listed in Table 4.

**Table 4:** Alternative set of elastic parameters: the bulk modulus *B*_0_ (in GPa), the factor of anisotropy *C*_*a*_, the shear modulus *G* (in GPa), and the Young’s modulus in two crystallographic directions *E*_100_ and *E*_111_ (in GPa).

Compound	B_0_		C_α_		G		E_100_		E_111_	
FeAl	164	−179	0.54	−0.53	81.5	−85.5	210	−221	333	−370
Fe_3_Al	153	−171	0.12	0.08	21	−9.5	60	−28	322	−270
Fe_3_AlC(sp)	216	−206	2.39	−2.47	175	−168.5	477	−397	217	−227
Fe_2_AlC	—	−195	—	−1.8	—	−173.5	—	−402	—	−256
FeMnAlC	—	−201	—	−1.94	—	−195	—	−446	—	−263
Mn_3_AlC	—	−214	—	−1.59	—	−175	—	−412	—	−285

The Fe_3_AlC phase showed a large bulk modulus and also a strong degree of anisotropy (*C*_*a*_ = 2.39 from the first method and 2.47 from the second method). This anisotropy was also showed by phase-field modelling of *κ*-carbide^9^ and also confirmed by experiments^11^. It is expected in metals, that *C*_*a*_ is smaller than 1 but these calculations showed that this value for *κ* was greater than 1. This led *E*_111_ to be smaller than *E*_100_. In addition, the degree of anisotropy for FeAl compound was small (*C*_*a*_ = 0.54) from the first method and 0.53 from the second method). This low degree of anisotropy was also in good agreement with the phase-field simulation of the B2 intermetallic compound^11^. Again, upon the substitution of Fe atoms by Mn ones, the various properties such as bulk modulus, shear modulus, and Young’s modulus increased confirming the experimentally observed increase in the hardness of the *κ* phase enriched by Mn atoms. This is supported by the experimental results (point 2 in the results section), wherein the *κ*-ferrite hardness was higher in the structures with higher Mn containing precipitates obtained at 700 °C compared to those obtained at 500 °C. In addition, the shear modulus of Fe_3_AlC was greater than that of FeAl or Fe_3_Al confirming the un-shearable nature of these precipitates that was observed experimentally.

## Conclusions

After annealing at temperatures between 500 and 1050 °C the hardness of micro- and nano-structural features found in Fe-15Mn-10Al-0.8C-5Ni and Fe-15Mn-10Al-0.8 C low-density steels were investigated experimentally through nano-indentation and computationally through *ab-initio* calculations. The hardness of micro-structures with B2 precipitates was measured through nano-indentation, whereas the hardness of nano-sized *κ* phase was evaluated through *ab-initio* calculations.

- The addition of Ni increased the hardness of the B2 phase due to the replacement of Ni-Al bonds over the Fe-Al bonds. In the presence of Ni in the alloy, the peak hardness increased from 7.2 GPa to 9 GPa. This was observed in both the austenite and the ferrite phases.
- The nano-sized cuboid *κ* and micron-sized B2 phases obtained in austenite when the Ni containing samples were annealed at 700 °C or lower temperature improved the hardness further in the austenite phase. The *γ* phase in samples annealed at higher temperatures (900–1050 °C), resulted in lower hardness due to the instability of nano-sized cuboid *κ*.
- The ferritic phase obtained in the Ni-containing alloys annealed at 900–1050 °C, produced a hard structure of 10 *GPa* due to the dispersion of nano-sized discs of B2 phase.
- Based on the first-principles calculations of different ordered structures, it was found that the insertion of a C atom only enhances the elastic constant in the *κ*-phase (${\rm{L}}{1^{\prime} }_{2}$) whereas it has a lesser effect on the Fe_3_Al−L1_2_phase. However, the presence of Mn atoms in the structure of Fe_3_AlC−κ increases its elastic constant. This was supported by the experimental results.

## Methods

### *ab-initio* calculations

In the first method, the slope of the acoustic branches in the [100] direction was used to calculate *C*_11_ and *C*_44_:

1 $${\nu }_{l}^{[100]}=\sqrt{\frac{{C}_{11}}{\rho }}$$

2 $${\nu }_{t}^{[100]}=\sqrt{\frac{{C}_{44}}{\rho }}$$

where ${\nu }_{l}^{[100]}$ and ${\nu }_{t}^{[100]}$ are the longitudinal and transverse velocity of sound in the compound, respectively. *ρ* is the density of the compound: for FeAl 5839, for Fe_3_Al 6612 and for Fe_3_AlC 6527 (all in kg/m^3^). [111] direction was used to calculate the value of *C*_12_:

3 $${\nu }_{l}^{[111]}=\sqrt{\frac{{C}_{11}+2{C}_{12}+4{C}_{44}}{3\rho }}$$

In the second method, a volume conserving orthorhombic distortion

4 $${\epsilon }=(\begin{array}{ccc}\delta  & 0 & 0\\ 0 & -\delta  & 0\\ 0 & 0 & \frac{{\delta }^{2}}{1-{\delta }^{2}}\end{array})$$

the associated change in total energy can be written as

5 $$E(\delta )=E(0)+({C}_{11}-{C}_{12})V{\delta }^{2}+O({\delta }^{4})$$

where *V* is the volume of the unit cell. This energy was solved to determine *C*_11_ and *C*_12_. For *C*_44_, a monoclinic distortion

6 $${\epsilon }=(\begin{array}{ccc}0 & \frac{\delta }{2} & 0\\ \frac{\delta }{2} & 0 & 0\\ 0 & 0 & \frac{{\delta }^{2}}{4-{\delta }^{2}}\end{array})$$

results in a total energy change as following:

7 $$E(\delta )=E(0)+\frac{1}{2}{C}_{44}V{\delta }^{2}+O({\delta }^{4})$$

For a tetragonal crystal structure, three additional independent elastic constant exists, namely *C*_13_, *C*_33_, and *C*_66_. The scheme presented in the ref.^40^ was used to determine these elastic constants for Fe_2_MnAlC and FeMn_2_AlC phases.

The practical parameters are calculated according to the following equations^40^:

8 $${B}_{0}=\frac{{C}_{11}+2{C}_{12}}{3}$$

9 $${C}_{a}=\frac{{C}_{11}-{C}_{12}}{2{C}_{44}}$$

10 $$G=\frac{{C}_{11}-{C}_{12}}{2}$$

11 $${E}_{100}=\frac{({C}_{11}+2{C}_{12})({C}_{11}-{C}_{12})}{{C}_{11}+{C}_{12}}$$

12 $${E}_{111}={(\frac{1}{{E}_{100}}+\frac{{C}_{11}-{C}_{12}-2{C}_{44}}{3{C}_{44}({C}_{11}-{C}_{12})})}^{-1}$$
